# High prevalence of vitamin D deficiency in Taiwanese patients with inflammatory bowel disease

**DOI:** 10.1038/s41598-024-64930-8

**Published:** 2024-06-18

**Authors:** Chen-Ta Yang, Hsu-Heng Yen, Pei-Yuan Su, Yang-Yuan Chen, Siou-Ping Huang

**Affiliations:** 1 Department of Post-Baccalaureate Medicine, College of Medicine, National Chung Hsing University, Taichung, 400 Taiwan; 2 Division of Gastroenterology, Changhua Christian Hospital, Changhua, 500 Taiwan; 3 Department of Hospitality Management, MingDao University, Changhua, 500 Taiwan

**Keywords:** Inflammatory bowel disease, Crohn’s disease, Vitamin D, Ulcerative colitis, Taiwanese population, Gastroenterology, Gastrointestinal diseases, Gastrointestinal system

## Abstract

Vitamin D deficiency is common in patients with inflammatory bowel disease (IBD). In this study, we aimed to evaluate the prevalence and risk factors of vitamin D deficiency in a Taiwanese IBD cohort. Vitamin D levels were checked in adult patients with IBD who were treated at Changhua Christian Hospital, a medical center in central Taiwan, from January 2017 to December 2023. The risk factors for vitamin D deficiency were evaluated. 106 adult IBD patients were included, including 20 patients with Crohn’s disease and 86 with ulcerative colitis. The median age at diagnosis was 39.2 years. The mean vitamin D level was 22.2 ± 8 ng/mL. Forty-five patients (42.5%) had vitamin D deficiency (vitamin D level < 20 ng/mL). Comparing patients with normal vitamin D levels and those with vitamin D deficiency after multivariate adjustment, female sex and early age at diagnosis were identified as statistically significant risk factors. We found a prevalence of 42.5% of vitamin D deficiency in the Taiwanese IBD population. Understanding this issue is essential for teaching patients and doctors about vitamin D deficiency screening and improving patient outcomes.

## Introduction

Inflammatory bowel diseases (IBD), including ulcerative colitis (UC) and Crohn’s disease (CD), are chronic inflammatory disorders of the gastrointestinal tract that require lifelong treatment to achieve sustained remission and to prevent disease flares. UC is a continuous disease involving the mucosal layer of the rectum to the colon, whereas CD involves the whole layer (transmural) of the gastrointestinal tract with a skipped pattern. Because of chronic inflammation of the bowel which interferences absorption of micronutrients^1^ and frequent steroid treatment in IBD patients, vitamin D deficiency is an important issue in this population. Vitamin D deficiency is associated with disease activity^2–8^ and results in decreased bone marrow density and fracture events in IBD patients^9–11^. The prevalence of vitamin D deficiency ranges from 22 and 63% according to different studies which use different reference value to define deficiency^2,3,7,8,12–15^. The etiologies of IBD are multi-factorial, comprising interactions between genes, the environment, the immune system, and microbiota^16,17^. Epidemiologic research has shown that IBD is more frequently diagnosed at higher altitudes^18^ and during winter^19^, suggesting a connection between its development and vitamin D deficiency. Traditionally, vitamin D plays a role in bone and mineral metabolism, but recent evidence shows that it also plays a role in the pathogenesis of several autoimmune disorders^20,21^ and IBD^22^, as well as serving as an immune-regulatory factor between the host immune system and gut microbiota^23,24^.

Globally, vitamin D deficiency, defined as a concentration < 20 ng/mL (50 nmol/L) according to the Endocrine Society guideline^25^, is common in the general population across all age groups^26^. Vitamin D deficiency is associated with many health problems such as chronic kidney disease^27–29^, diabetes mellitus^20,30^, cardiovascular disease^31^, cerebrovascular disease^32^, certain types of malignancy^33^, and autoimmune disease^20,21^. However, it remains unclear whether a lack of vitamin D leads to the development of these disorders or whether it is a symptom. Vitamin D has been shown to have an immune modulatory role and may be implicated in the etiology of diseases. Moreover, a lack of vitamin D frequently arises because of chronic disorders. The major source of vitamin D originates from skin production by exposure to ultraviolet B radiation from sunlight, with only a small part obtained from food. Many factors are associated with vitamin D deficiency, including insufficient sunlight exposure, reduced dietary sources, lack of physical activity, chronic kidney disease that reduces the renal transformation of active vitamin D, and malabsorption or intestinal inflammation^22,29,34–36^.

Vitamin D deficiency is a recognized problem among patients with IBD as well as the general population^2,3,5,8,37^. A meta-analysis of 14 studies by Rita et al.^38^, comprising 1891 patients (938 patients with IBD and 953 controls), showed that patients with IBD had 64% higher odds of vitamin D deficiency than controls (OR: 1.64, 95% CI 1.30–2.08). Nonetheless, most reported cases were from regions with a high prevalence of IBD, and data from the Asian population is limited. In Taiwan, the incidence and prevalence of IBD are increasing^39^. Therefore, it is crucial to determine the prevalence of vitamin D insufficiency in this growing population. The aim of this study was to examine the prevalence and risk factors of vitamin D insufficiency in a Taiwanese IBD cohort.

## Materials and methods

### Study design and patients

This study was conducted at a tertiary medical center in central Taiwan, enrolling adult IBD patients who participated in a prospective observational registry study. Informed consent was obtained from all subjects who participated this study. Patients who had check-ups for vitamin D concentrations between January 2017 and December 2023 were included in this analysis. IBD was diagnosed through clinical symptoms, signs, an endoscopic image, and histological confirmation with a catastrophic illness certificate from Taiwan Health Insurance. The inclusion criteria included a diagnosis of either Crohn’s disease or ulcerative colitis certified by the Ministry of Health and Welfare of Taiwan. The exclusion criteria included patients aged < 20 years at the date of vitamin D concentration check-up. Demographic, clinical (e.g., disease activity, extent of GI tract involvement, and modality of treatment), and laboratory data were collected. The modality of treatment included steroids, 5-aminosalicylic acid (5-ASA), biologics, and azathioprine (AZA). Recent use of medication is defined as within 3 months of measurement of vitamin D. Laboratory data included white blood cell (WBC) count, hemoglobin (Hb), platelet count, albumin, and c-reactive protein (CRP). Risk factors for vitamin D were evaluated from the patients’ medical records. The study was approved by the Institutional Review Board (IRB) of Changhua Christian Hospital (IRB number: CCH IRB No. 181014, date of approval: November 26, 2018).

### Laboratory studies

The serum vitamin D concentration (25-OH vitamin D total) was quantitatively measured using the electrochemiluminescence method via a Cobas® immunoassay analyzer from Roche Diagnostics, Mannheim, Germany. Before May 15, 2020, we used Elecsys Vitamin D Assay, and after May 15, 2020, we used Elecsys Vitamin D Total II, which is a second-generation assay. The results are expressed in nanograms per milliliter (ng/mL). Vitamin D deficiency is defined as a concentration < 20 ng/mL, while a normal level is defined as ≥ 20 ng/mL, including insufficient (between 20 and 29 ng/mL) and sufficient (≥ 30 ng/mL) vitamin D levels.

### Definition of remission

The definition of clinical remission of CD is a Crohn’s disease activity index (CDAI) of < 150^40^. The CDAI is composed of eight clinical or laboratory variables with different weights and ranges from 0 to 600. The definition of clinical remission in UC is a Mayo score of < 3 points (including 0, 1, or 2)^41^. The Mayo score consists of four parts of sub-scores (0–3), including rectal bleeding, stool frequency, physician assessment, and endoscopy appearance, with a total possible score of 0–12.

### Statistical analysis

Categorical variables are presented as numbers and percentages. Continuous data are expressed as mean and standard deviation or as the median and interquartile range (IQR) for normally and non-normally distributed data, respectively. The distribution of continuous variables was examined by one-sample Kolmogorov–Smirnov test. Categorical variables were compared by the chi-square test or the Fisher’s exact test; continuous variables were compared by Student’s t-test or the Mann–Whitney U-test, as appropriate. Multivariable logistic regression analysis was performed to identify factors associated with vitamin D deficiency. In the multivariate adjustment model with backward elimination, data were adjusted for age, duration from disease onset to vitamin D measurement, sex, clinical remission, treatments (e.g., steroids, 5-ASA, biologics, and AZA), laboratory data (WBC count, Hb, platelet count, albumin, and CRP), IBD subtypes, and abdominal surgery. Statistical analysis was performed using IBM SPSS Statistics for Windows, Version 22.0 (IBM Corp., Armonk, NY). *P* values < 0.05 were considered statistically significant.

## Results

**Figure 1:**
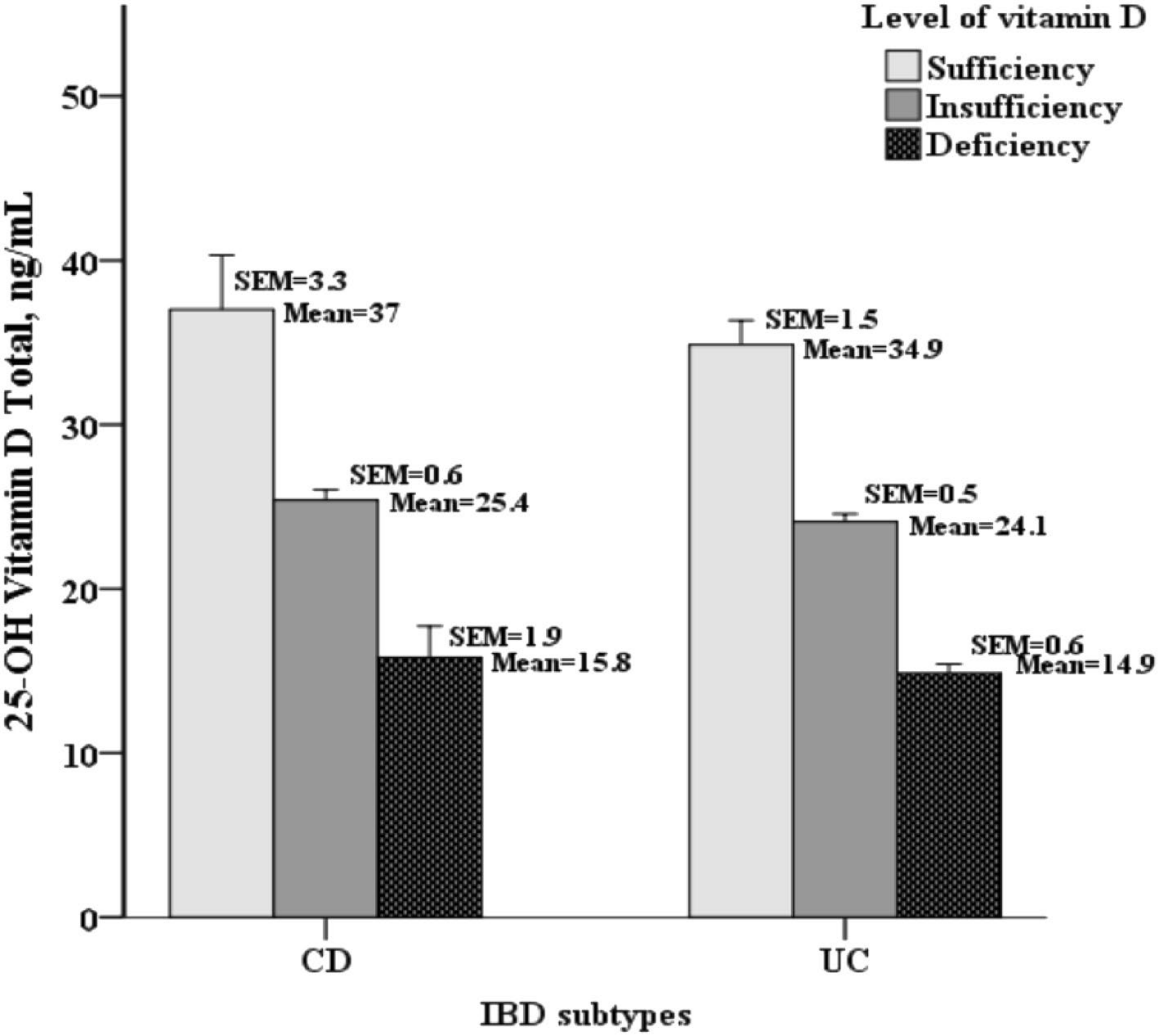
Mean and standard error of mean (SEM) of the total 25-OH vitamin D among patients with CD and those with UC (classified as sufficient if vitamin D ≥ 30 ng/mL, insufficient 20–29 ng/mL, and deficient < 20 ng/mL).

A total of 106 adult patients with IBD who fulfilled the inclusion criteria were included, including 20 patients with CD and 86 with UC. The median age at diagnosis was 39.2 years, with a predominant male population (57.5%). The comparison of baseline characteristics between patients with CD and those with UC are shown in Table 1. The mean vitamin D concentration was 22.2 ± 8 ng/mL in the whole study population, 24.7 ± 7.4 ng/mL in patients with CD, and 21.6 ± 8.1 ng/mL in patients with UC. According to the level of vitamin D, 45 patients (42.5%) had vitamin D deficiency, 44 patients (41.5%) had vitamin D insufficiency, and 17 patients (16.0%) had a sufficient vitamin D level (Table 1). According to the subgroups of vitamin D sufficiency, insufficiency, and deficiency, the mean 25-OH vitamin D total was higher in patients with CD than those with UC (sufficiency: 37[3.3] vs. 34.9[1.5], insufficiency: 25.4[0.6] vs. 24.1[0.5], deficiency: 15.8[1.9] vs. 14.9[0.6]) as shown in Fig. 1.

**Table 1:** Baseline characteristics of the IBD population (comparisons between patients with CD and UC).

Variables	All patients (n = 106)	CD (n = 20)	UC (n = 86)	*P* value
Age at diagnosis, year, median (IQR)	39.2 (30.2–52.2)	43.6 (27.2–57.2)	39.2 (30.4–50.8)	0.55
Duration from disease onset to vitamin D measurement, year, median (IQR)	2 (1–7)	3 (0.5–9.5)	2 (1–7)	0.794
Sex, male, n (%)	61 (57.5%)	16 (80.0%)	45 (52.3%)	0.024
Clinical remission, n (%)	56 (52.8%)	11 (55.0%)	45 (52.3%)	0.829
Recent steroids, n (%)	24 (22.6%)	5 (25.0%)	19 (22.1%)	0.772
Recent 5-aminosalicylic acid, n (%)	82 (77.4%)	14 (70.0%)	68 (79.1%)	0.385
Recent biologics, n (%)	4 (3.8%)	2 (10.0%)	2 (2.3%)	0.161
Recent azathioprine, n (%)	10 (9.4%)	5 (25.0%)	5 (5.8%)	0.02
Laboratory data				
White blood cell count, 10^3^/μL, mean ± SD	7.2 ± 2	7.4 ± 1.8	7.2 ± 2	0.711
Hemoglobin, g/dL, median (IQR)	13.7 (12.4–14.5)	13.8 (11.6–14.3)	13.7 (12.5–14.6)	0.608
Platelet count, 10^3^/μL, mean ± SD	298.8 ± 95.1	325.3 ± 86.2	292.9 ± 96.5	0.182
Neutrophil, %, mean ± SD	65.7 ± 9.2	68.7 ± 8.6	65 ± 9.2	0.109
Lymphocyte, %, median (IQR)	24.5 (18.1–29.4)	21.5 (14.1–24.7)	25.2 (19.4–29.8)	0.068
Albumin, g/dL, median (IQR)	4.4 (4.1–4.5)	4.3 (3.9–4.5)	4.4 (4.1–4.5)	0.368
C-reactive protein, mg/dL, median (IQR)	0.13 (0.07–0.51)	0.17 (0.07–1.72)	0.13 (0.06–0.47)	0.203
25-OH vitamin D total, ng/mL, mean ± SD	22.2 ± 8	24.7 ± 7.4	21.6 ± 8.1	0.111
Level of vitamin D, n (%)				0.129
Deficiency (< 20 ng/mL)	45 (42.5%)	5 (25.0%)	40 (46.5%)	
Insufficiency (20 –29 ng/mL)	44 (41.5%)	12 (60.0%)	32 (37.2%)	
Sufficiency (≥ 30 ng/mL)	17 (16.0%)	3 (15.0%)	14 (16.3%)	
Abdominal surgery, n (%)	6 (5.7%)	6 (30.0%)	0 (0.0%)	< 0.001
CD location, n (%), L1 /L2 /L3 /L4*	–	7 (35.0%)/2 (10.0%)/10 (50.0%)/1 (5.0%)	–	
CD behavior, n (%), B1/B2/B2p B3/B3p#	–	6 (30.0%)/9 (45.0%)/1 (5.0%)/3 (15.0%)/1 (5.0%)	–	–
UC location, n (%), E1 /E2 /E3$	–	–	23 (26.7%)/30 (34.9%)/33 (38.4%)	–

*L1 for disease in the terminal ileum, L2 for disease in the colon, L3 for ileocolonic disease, and L4 for concomitant upper gastrointestinal diseases.

#B1 non-stricturing and non-penetrating, B2 stricturing disease, B2p stricturing disease with perianal fistulas, B3 penetrating disease, and B3p penetrating disease with perianal fistulas.

$E1 proctitis, E2 left-sided colitis, and E3 pancolitis.

*CD* Crohn’s disease, *UC* ulcerative colitis, *SD* standard deviation, *IQR* interquartile range, *μL* microliter, *g/dL* gram per deciliter, *mg* milligram, *25-OH* 25-hydroxy, *ng/mL* nanogram per milliliter.

Among the whole study population, 56 patients (52.8%) remained in clinical remission. The patients with CD presented a higher percentage of the stricturing type (45%), followed by non-stricturing and non-penetrating type (30%), penetrating type (15%), and perianal fistula (10%). Additionally, among patients with UC, 38.4% of patients had extensive colitis, 34.9% had left-side colitis, and 26.7% had proctitis. For recent IBD medications, including steroids, 5-ASA, and biologics, there was no significant difference between the CD and UC groups, but more patients with CD had recent AZA (25% in the CD group and 5.8% in the UC group). The laboratory data showed no significant difference between the CD and UC groups. Six patients had abdominal surgery, all of whom were in the CD group.

Table 2 shows the comparisons between the groups with a normal vitamin D level and those with a deficiency. The vitamin D deficient group had less males (37.8%) (*p* < 0.001). Among several laboratory parameters, significantly increased leukocyte counts (7.8 ± 2.1 vs. 6.8 ± 1.8 *10^3^/μL, *p* = 0.012) and significantly decreased Hb levels (13 [11.6–14.3] vs. 14 [13–14.9] g/dL, *p* = 0.006) were found in the vitamin D deficient group. Moreover, there was a trend toward younger age at diagnosis in the group with vitamin D deficiency (35.5 [28.2–48.9] vs. 43.3 [30.9–54.1], *p* = 0.060), but no significant difference in the duration from disease onset to vitamin D measurement between the two groups (median 3 and 1 years for patients with normal and deficient vitamin D levels, respectively; *p* = 0.422).

**Table 2:** Baseline characteristics of the IBD population (comparisons between the groups with and without vitamin D deficiency).

Variables	All patients (n = 106)	Without deficiency (n = 61)	With deficiency (n = 45)	*P* value
Age at diagnosis, year, median (IQR)	39.2 (30.2–52.2)	43.3 (30.9–54.1)	35.5 (28.2–48.9)	0.060
Duration from disease onset to vitamin D measurement, year, median (IQR)	2 (1–7)	3 (1–9)	1 (1–6)	0.422
Sex, male, n (%)	61 (57.5%)	44 (72.1%)	17 (37.8%)	< 0.001
Clinical remission, n (%)	56 (52.8%)	35 (57.4%)	21 (46.7%)	0.275
Recent steroids, n (%)	24 (22.6%)	14 (23.0%)	10 (22.2%)	0.929
Recent 5-aminosalicylic acid, n (%)	82 (77.4%)	46 (75.4%)	36 (80.0%)	0.577
Recent biologics, n (%)	4 (3.8%)	1 (1.6%)	3 (6.7%)	0.309
Recent azathioprine, n (%)	10 (9.4%)	8 (13.1%)	2 (4.4%)	0.184
Laboratory data				
White blood cell count, 10^3^/μL, mean ± SD	7.2 ± 2	6.8 ± 1.8	7.8 ± 2.1	0.012
Hemoglobin, g/dL, median (IQR)	13.7 (12.4–14.5)	14 (13–14.9)	13 (11.6–14.3)	0.006
Platelet count, 10^3^/μL, mean ± SD	298.8 ± 95.1	294.9 ± 103.1	304 ± 84.4	0.632
Neutrophil, %, mean ± SD	65.7 ± 9.2	65.3 ± 9.7	66.2 ± 8.6	0.645
Lymphocyte, %, median (IQR)	24.5 (18.1–29.4)	24.3 (15.8–30.2)	25 (20.8–28.4)	0.941
Albumin, g/dL, median (IQR)	4.4 (4.1–4.5)	4.4 (4.1–4.5)	4.2 (4–4.5)	0.223
C-reactive protein, mg/dL, median (IQR)	0.13 ( 0.07–0.51)	0.12 (0.06–0.53)	0.18 (0.07–0.51)	0.592
25-OH vitamin D total, ng/mL, mean ± SD	22.2 ± 8	27.5 ± 6	15 ± 3.5	
IBD subtypes, n (%)				0.080
Ulcerative colitis	86 (81.1%)	46 (75.4%)	40 (88.9%)	
Crohn’s disease	20 (18.9%)	15 (24.6%)	5 (11.1%)	
Abdominal surgery, n (%)	6 (5.7%)	4 (6.6%)	2 (4.4%)	1.000

*SD* standard deviation, *IQR* interquartile range, *μL* microliter, *g/dL* gram per deciliter, *mg* milligram, *25-OH* 25-hydroxy, *ng/mL* nanogram per milliliter.

Table 3 illustrates the risk factors related to vitamin D deficiency. The results revealed no significant difference between patients with and without vitamin D deficiency in terms of the medications, percentage of patients in clinical remission, or abdominal surgery for IBD. Increased age at diagnosis (OR: 0.95, 95% CI 0.92–0.99, *p* = 0.007) and male sex (OR: 0.23, 95% CI 0.09–0.63, *p* = 0.004) are linked to a considerably lower odds ratio after multivariate adjustment. In other words, early age at diagnosis and female sex were identified as statistically significant risk factors of vitamin D deficiency. Additionally, an increased leukocyte count is associated with increased risk of vitamin D deficiency (OR 1.34, 95% CI 1.01–1.77, *p* = 0.042).

**Table 3:** Risk factors related to vitamin D deficiency.

Variables	Crude logistic model		Adjusted logistic model*	
	Crude OR (95% CI)	*P* value	Adjusted OR (95% CI)	*P* value
Age at diagnosis	0.97 (0.95, 1)	0.052	0.95 (0.92, 0.99)	0.007
Duration from disease onset to vitamin D measurement	0.97 (0.91, 1.04)	0.391	0.93 (0.86, 1.01)	0.086
Sex (male)	0.23 (0.1, 0.53)	0.001	0.23 (0.09, 0.63)	0.004
Clinical remission	0.65 (0.3, 1.41)	0.276	–	–
Recent steroids	0.96 (0.38, 2.41)	0.929	–	–
Recent 5-aminosalicylic acid	1.3 (0.51, 3.32)	0.577	–	–
Recent biologics	4.29 (0.43, 42.63)	0.214	–	–
Recent azathioprine	0.31 (0.06, 1.53)	0.150	–	–
White blood cell count	1.31 (1.05, 1.63)	0.016	1.34 (1.01, 1.77)	0.042
Hemoglobin	0.73 (0.58, 0.92)	0.008	–	–
Platelet count	1 (1, 1.01)	0.629	–	–
Neutrophil	1.01 (0.97, 1.05)	0.642	–	–
Lymphocyte	1 (0.95, 1.05)	0.953	–	–
Albumin	0.6 (0.22, 1.63)	0.319	–	–
C-reactive protein	1.1 (0.84, 1.44)	0.486	–	–
IBD subtypes			–	–
Ulcerative colitis	1 (Reference)	–	–	–
Crohn’s disease	0.38 (0.13, 1.15)	0.087	–	–
Abdominal surgery	0.66 (0.12, 3.79)	0.644	–	–

*The first step 1 in model was adjusted for age, duration from disease onset to vitamin D measurement, sex, clinical remission, treatments (e.g., steroids, 5-ASA, biologics, and AZA), laboratory data (WBC count, Hb, platelet count, albumin, and CRP), IBD subtypes, and abdominal surgery.

*OR* odds ratio, *CI* confidence interval, *IBD* inflammatory bowel disease.

## Discussion

Our study is the first to report the prevalence and risk factors of vitamin D deficiency among the Taiwanese IBD population. Our study revealed a mean vitamin D concentration 22.2 ± 8 ng/mL and 42.5% of our population with vitamin D deficiency. In a study of vitamin D deficiency among a healthy adult population in northern Taiwan, the mean vitamin D concentration was 28.9 ng/mL, and 22.4% of the study population had vitamin D deficiency^42^. Several co-factors, including diet, outdoor activity, use of sunscreen lotion, percentage of sunny/rainy days, female sex, urban living, educational status, vitamin D supplementation, and chronic diseases, are known to influence the prevalence of vitamin deficiency. The study of the healthy general population of northern Taiwan showed that the predictors of vitamin D deficiency were female sex, lower age, high educational status, living in an urban area, and physical inactivity, while our study showed that female sex and early age at diagnosis were risk factors.

Vitamin D is mainly synthesized in the skin upon exposure to sunlight, and 20% is obtained from dietary sources^26,30,36^. Previous studies found a high prevalence of vitamin D deficiency among patients with IBD^26,43,44^. Additionally, the Asian population with dark skin pigmentation may have reduced sunlight penetration, which can have a negative effect on vitamin D synthesis^13,45^. Moreover, vertebral fractures are more common in patients with IBD than in the healthy population^9^. Before beginning steroid medication, it is crucial to understand the status of vitamin D deficiency. To prevent low bone mineral density, vitamin D monitoring and supplementation are suggested in patients with IBD with active disease or those who are treated with steroids^46^. The prevalence of vitamin D deficiency in the IBD population ranged from 40%^47^ to 92%^48^ in Korea, 60–100% in Japan^10^, and 42% in the present study. Thus, although it is vital to determine the prevalence of vitamin D deficiency in the Asian population, this is not currently addressed in the Asian IBD practice recommendations^49–51^.

Vitamin D receptors are found in nearly all types of immune cells, highly expressed in the small intestine and colon and involved in both innate and adaptive immune systems. Vitamin D also plays an important role in intestinal homeostasis where it serves to maintain the integrity of the intestinal barrier^52^. However, it remains unclear whether vitamin D deficiency is a contributing factor to the pathogenesis of IBD^53,54^ or a consequence of malabsorption from intestinal damage^55^. Previous studies have reported lower vitamin D levels in women^56,57^, and our study showed that female sex is a risk factor. However, no such correlation is shown in other studies^2,3,8,58^. The study by Alex Ulitsky et al.^2^ showed that older age and older age at diagnosis were associated with vitamin D deficiency. Our study showed that younger age at diagnosis is a risk factor for vitamin D deficiency. Moreover, younger age at disease onset has been shown to be an unfavorable prognostic factor for IBD^59,60^, which requires more medical attention in the future. Our findings indicate the necessity of vitamin D screening in our young population^51,61^. Meta-analysis showed vitamin D supplementation effectively improves the vitamin D concentration and decreases CRP level in adult^43^ and pediatric IBD patients^62^. However, the prevalence of untreated vitamin D deficiency is 82% in IBD patients^63^ which disclosed clinical overlook of the issue of vitamin D deficiency. Society guidelines recommend supplementation of vitamin D is recommended for those with documented deficiency^49–51^ to prevent osteoporosis during disease treatment. Additionally, such supplementation is safe and has a positive effect on disease outcomes, which is particularly important for the pediatric population when therapeutic options for IBD are often restricted^43,44,64^.

Vitamin D levels differ between patients with CD and those with UC. Due to the involvement or resection of the small intestine in CD, some studies have found lower vitamin D levels among patients with CD than among those with UC^7,12^. However, in our study, the mean vitamin D concentration was found to be lower in patients with UC than in those with CD, although not significantly (UC 21.6 ± 8.1 ng/mL, CD 24.7 ± 7.4 ng/mL). This may be attributed to the small percentage of patients with CD in our study. Vitamin D deficiency is linked to disease activity^5,37,65^. Indeed, a previous meta-analysis of 27 studies from Gubatan et al.^44^, comprising 8316 patients with IBD, summarized that low vitamin D level is significantly associated with disease activity (OR: 1.53, 95% CI 1.32–1.77). In addition to clinical evaluation, studies have demonstrated an inverse relationship between vitamin D level and inflammatory markers, such as leukocyte counts, CRP, and fecal calprotectin, in the IBD population^4,12,66^. In our study, the group with vitamin D deficiency had a significantly higher leukocyte count (7.8 ± 2.1 vs. 6.8 ± 1.8 *10^3^/μL, *p* = 0.012); however, there was no significant difference in CRP levels. The group with vitamin D deficiency had a significantly lower Hb level compared to the control group in our research (13 [11.6–14.3] vs. 14 [13–14.9] g/dL, *p* = 0.006), which may reflect the severity of bowel damage. Moreover, in our study, a lower percentage of patients remained in clinical remission in the group with vitamin D deficiency (46.7% vs. 57.4%, *P* = 0.275), although the difference was not statistically significant.

Our investigation has several limitations that warrant discussion. First, the number of cases was relatively low, particularly in the CD group. This limited the currently low prevalence of IBD population in our region^67,68^. Second, although this is a prospective and observational study of patient medical records, undergoing a vitamin D check-up and receiving subsequent treatment were not mandatory for participation. Additionally, we did not investigate the outcomes for patients after vitamin D treatment. However, determining the impact of such treatment is challenging, as supplements are commonly sold over-the-counter, making it difficult to monitor their intake retrospectively. Additionally, the measurement of vitamin D and treatment of its insufficiency are currently not reimbursed by our national health insurance. Indeed, only two-thirds of our IBD population reported having undergone a check-up of vitamin D concentration in our institution^68^. Since therapies and tests to measure vitamin D levels require out of pocket money and given that not all patients are willing to undergo screening, it is critical that more people become aware of the significant lack of vitamin D that this population is experiencing, and the current study represents an important step in this direction. It is necessary to perform further research to analyze the food pattern, sun exposure, and vitamin D supplementation pattern and clinical response in this community to ensure a better understanding of the link between vitamin insufficiency and this population. Third, we did not record the frequency of sun exposure, the amount of vitamin D-rich foods consumed, or outdoor exercise among the study population, all of which could have been confounding factors for vitamin D levels. Forth, there is no healthy control in our study to compare the vitamin D level.

## Conclusion

The current study concluded that the Taiwanese population with IBD had a prevalence of 42.5% of vitamin D deficiency. Understanding this condition is crucial to raising patients' and doctors' awareness of the need to screen for vitamin D deficiency and enhance patient outcomes.

## Data Availability

The datasets generated and/or analyzed during the current study are not publicly available, but these may be requested from the corresponding author, upon reasonable request.
